# Feasibility and validation of a novel mobility monitoring sensor in hospitalized patients: A prospective cohort study

**DOI:** 10.1017/cts.2025.10110

**Published:** 2025-07-24

**Authors:** Samuel Smith, Leah Steckler

**Affiliations:** George Washington University School of Medicine and Health Sciences, Washington, DC, USA

**Keywords:** Biomedical technology, mobility limitation, patient monitoring, pressure ulcer, secondary disease prevention, wireless sensing

## Abstract

**Background:**

Hospital-acquired pressure injuries (HAPIs) are a preventable source of patient harm, contributing to morbidity, mortality, and billions in healthcare costs. Risk assessment tools rely on subjective evaluation and may not accurately capture real-time mobility. Existing technologies have not been widely adopted and have failed to significantly reduce HAPI rates. Our study explores the feasibility of a novel, wireless mattress-attachable motion sensor designed for continuous mobility monitoring in hospitalized patients.

**Methods:**

Sensor accuracy was first validated against video analysis in three healthy volunteers. A single-arm prospective cohort study was then conducted in hospitalized patients. A motion sensor was attached to each patient’s bed to continuously record movement. Sensor-derived mobility data were compared with nursing-assessed mobility scores and other patient characteristics. Simulated immobility alerts were generated based on periods of inactivity.

**Results:**

The sensor’s movement detection strongly correlated with video-based analysis in three healthy volunteers (*r* = 0.89, 95% CI [0.51, 0.99]). Forty-seven patients were enrolled with an average of 9.7 movements/hour and average recording duration of 22.9 hours. No significant differences in age, comorbidities, or nursing mobility scores were observed between high- and low-movement groups. Simulated immobility alerts identified 15 patients who would have triggered a notification, predominantly those with lower movement and BMI.

**Conclusions:**

The sensor system provides objective mobility data and overcomes limitations of current assessment tools. These findings support its potential role in pressure injury prevention and highlight key areas for future clinical integration.

## Introduction

Hospital acquired pressure injuries (HAPIs) are seen worldwide. Estimates of prevalence in the United States vary considerably depending on care setting. One study showed the prevalence of HAPIs to be near 2.6% in the inpatient setting with a higher prevalence in the ICU compared to med-surg units [1]. Other studies show a prevalence of 0.4% – 38% for hospitals [2]. Costs related to HAPIs are estimated to exceed $26.8 billion annually and nearly $11,000, on average, per patient that develops a HAPI [3]. The cost of HAPIs is often not reimbursed due to regulations set by healthcare payers, particularly the Centers for Medicare & Medicaid Services (CMS). CMS, followed by many private insurers, classifies HAPIs as a “never event,” meaning they are considered preventable with proper care and protocols [4]. As such, in addition to the patient-related morbidity and mortality associated with HAPIs, there is a strong financial incentive in health care settings to prevent this adverse event.

Pressure injuries develop when sustained pressure is applied to the same area of skin for an extended period. Age related skin changes, nutritional status, chronic disease, and local moisture can all contribute to pressure injury development. In addition to these factors, immobility has been identified as the greatest risk factor [5–7]. To mitigate risk, clinicians reposition at-risk patients at least every four hours, as recommended by medical protocols [8]. Despite the widespread implementation of these protocols, pressure injuries remain prevalent due to several factors which make it difficult to adequately assess mobility in the hospital setting [9]. Inadequate assessment of pressure injury risk is likely due to a confluence of several factors including subjectivity of assessment tools, nurse training variability, changing mobility status, and staff shortages [10,11].

One widely used assessment tool is the Braden scale, which evaluates a patient’s risk of developing a pressure injury based on six subscales: sensory perception, moisture, activity, mobility, nutrition, and friction and shear [12]. Each subscale is scored from 1 to 4, with lower scores indicating greater vulnerability. Typically, scores below 18 suggest an increased risk of pressure injury [13]. The subjective nature of the Braden scale introduces variability due to differences in nursing staff experience, training, and clinical judgment. Several studies have called into question the efficacy of the Braden score for predicting pressure injury risk [14–16].

Advancements in sensor technology offer an opportunity to enhance the accuracy and consistency of immobility assessments. There are several existing technologies available for tracking patient positioning in bed for the purpose of pressure injury prevention, including body attachable sensors and pressure distribution mats. Pressure mats display pressure distribution beneath patients and can alert providers when intervention is needed. Body attachable motion sensors are placed on a patient’s chest to wirelessly monitor body position and signal when repositioning is required. Like the device used in this study, several of these technologies, such as the Leaf Patient Monitoring System and PRESENSE’s wearable sensor incorporate angle thresholds to determine repositioning events [17,18]. Despite their utility, both technologies have drawbacks. Pressure mats have been found to increase the risk of pressure injury development, likely by increasing the local pressure at the interface between the skin and support surface [19]. Body attachable sensors require adhesive that can damage the skin and have shown low compliance by patients due to discomfort [20]. Further, equipment attached to a patient can be considered a tether and may contribute to delirium in at-risk populations. Newer technologies under development use load cells underneath the wheels of hospital beds to assess patient movement [21]. While this technology is promising, it requires multiple sensors and may be complex and expensive to retrofit existing hospital beds.

**Figure 1.**
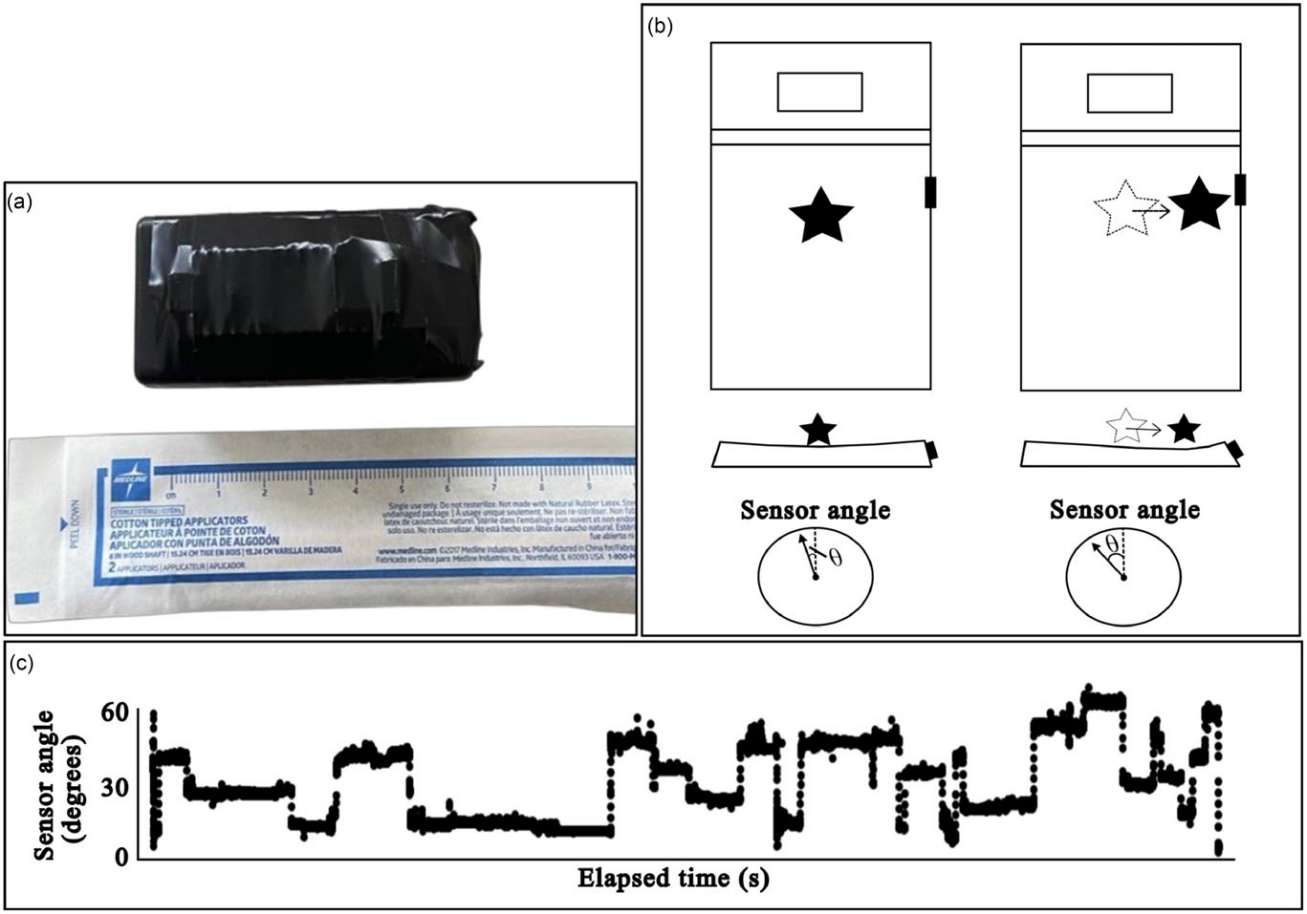
Angle based motion sensor diagram. (a) the device consists of a single 7 X 4 X 2 cm wireless sensor securely attached to the side of a patient’s mattress. (b) sensor measures the angle of deflection in the mattress caused by body movements. The sensor is represented by the black shaded rectangle (not drawn to scale). The sensor angle () increases when there is a shift in body weight (e.g. rolling over), where the patients center of mass is represented by a star. (c) changes in patients’ movement can be tracked by measuring changes in angle over time, represented by the step function shown from a patient enrolled in the study. 30 hours of data from patient 2 are shown in the figure.

With these constraints in mind, the research team has developed a novel motion sensor system designed to address the limitations of current assessment methods (Figure 1) [22]. The sensor uses an accelerometer to measure changes in the angle of the bed mattress as the patient changes position. It sends angle information at second intervals via Bluetooth to a bedside computing unit that processes the data and uses an algorithm to detect changes in position. This processing unit will also be used to generate alerts when patients are immobile for an extended period, though this feature is not yet implemented. Importantly, the sensor filters out minor, non-relevant disturbances while accurately detecting significant changes in body position.

Preliminary testing has demonstrated the sensor’s capability to reliably distinguish meaningful movements from incidental activity, making it a promising tool for clinical use. In this study the sensor was validated by comparing movement rates from video analysis to the novel sensor in a controlled setting. Then a prospective observational study was performed with the aims to evaluate the feasibility and reliability of this motion sensor system and compare its measurements to several patient-specific factors in hospitalized patients. The primary of objective of this study is to evaluate the feasibility and reliability of a novel mattress-based motion sensor for monitoring patient mobility in the hospital setting. The secondary objective is to compare the sensor-derived mobility data with established clinical risk factors and nursing assessments related to pressure injury risk. By integrating objective motion data, the research seeks to improve clinical practices and ultimately reduce the incidence of pressure injuries.

## Methods

Sensor Fabrication and Algorithm Implementation Sensors were fabricated using Arduino Nano 33 BLE microchips with built in inertial measurement unit and low-energy Bluetooth capabilities. The chip was mounted on a custom plastic housing that accommodated a 9-volt battery. They were programmed to transmitted 3-axis angular velocity, acceleration, and absolute angle readings every second. A bedside processing unit was fabricated using an Arduino Nano 33 BLE chip, liquid crystal display screen, secure digital (SD) card, and rechargeable battery pack. These components were soldered and sealed in an Acrylonitrile Butadiene Styrene enclosure to ensure stability and minimize electrical interference. This unit was programmed to connect with the bedside sensor via Bluetooth and read the sensor values. Bedside sensor *X*-axis angle was stored as “angle” and *Y*-axis angle was stored as “pitch.” The processing unit implemented an algorithm that analyzed angle, pitch, and elapsed time to determine patient movements. Thresholds of angle change greater than 2 degrees, pitch change of greater than 2 degrees, and elapsed time of 10 s were used. Thus, the device registered a position change only when both angle and pitch shifted by more than 2 degrees and remained changed for a minimum of 10 s. Elapsed time was used to ensure that movements were sustained and not transient. These thresholds were selected based on preliminary experiments with the sensor, with the goal of filtering out insignificant movements that did not constitute change in position within the bed. All data was stored in the SD card of the bedside unit for processing and analysis.

### Sensor validation

Three volunteers were recruited, and their movement was monitored using both video (captured on a GoPro Hero) and the motion sensor described above. They were given a prescribed set of movements to follow, including rolling left and right, sitting up, and getting out of bed. This was followed by a period of free movement time in the bed where the volunteers were instructed to move as they desired. Video analysis was performed using a custom Python (version 3.13.1) script that utilizes OpenCV and MediaPipe’s pose estimation framework to detect and track key body landmarks, including the shoulders, hips, and nose. The script processed each video frame, extracting pose data and calculating shifts in the body’s center of mass over time. A movement event was registered when a significant repositioning was detected, filtering out minor adjustments using a predefined movement threshold of 2.5% of the video frame width and a minimum sustained movement duration of 15 frames. The total number of repositioning events was recorded and compared to sensor-derived movement data. The movement data recorded from both the video analysis and sensor measurements were visualized using Matplotlib. To quantitatively assess the agreement between the two measurement techniques, Pearson correlation coefficients (r) were calculated individually for each of the three healthy validation participants. Movement counts from each method were first aggregated into sixty-second time bins, then merged by patient ID and timestamp. To summarize overall agreement, Fisher’s z-transformation was applied to each individual correlation coefficient, allowing computation of a 95% confidence interval around the average r value. The inverse z-transformation was used to report the final mean Pearson correlation and its confidence interval in r space.

### Patient recruiting and data collection

This study was conducted on inpatient medical and surgical floors at George Washington University Hospital from October 2024, to January 2025. Patients aged 18–100 years without movement disorders, pregnancy, or active pressure injuries on admission were included. Movement disorders could introduce abnormal motion unrelated to standard mobility, pregnancy may affect weight distribution and body movement, and existing pressure injuries could confound the evaluation of risk factors and alert generation.

#### Patient demographics

The patients in the study had an even gender distribution. The average age was 63 years, ranging from 24 to 97 years. The average body mass index (BMI) was 29.5 kg/m2, with a range of 19.8 to 48.9 kg/m2. Admitting diagnoses were diverse, including conditions such as adrenal crisis, pneumonia, heart failure exacerbation, lumbar radiculopathy, and viral myocarditis. Patients had an average of 8.8 comorbid chronic diagnoses based on ICD code. They were mostly admitted to the general medicine and cardiology services. A detailed summary of the study cohort’s demographics and clinical characteristics is provided in Table 1.

**Table 1.** Demographics and clinical characteristics of study cohort (n = 44)

Characteristic	Category	*n*	%	Mean	Range	SD
Age				62.9	73	18.8
Sex	Male	22	50.0			
	Female	22	50.0			
^a^Number of comorbid chronic diagnoses				8.8	29	6.6
Admitting service	General medicine	21	47.7			
	Cardiology	13	29.5			
	Surgery	5	11.4			
	Pulmonology	4	9.1			
	Neurology	1	2.3			
BMI (kg/m^2^ **)**				29.5	29.2	6.9

*Note*: Age, comorbid chronic diagnoses, and BMI are presented as mean with standard deviation (SD) and range. Ranges were calculated as the difference between the maximum and minimum values for each characteristic. Sex is reported as number (n) and percentage of cohort. Service distribution is presented as number on each service and percent relative to entire cohort.

a Number of comorbid chronic diagnoses per ICD code.

#### Data collection

Patients were recruited in the Emergency Department and provided informed consent prior to admission. A single motion sensor was affixed to the side of their hospital bed once they were admitted to the floor. Sensor angle, pitch, and 3-axis acceleration were measured by the sensor every 1 s. This data was transmitted via Bluetooth to a nearby central processing unit that stored the data and computed movements using an algorithm that relies on time and changes in angle and pitch. Braden scale scores were assessed bedside daily to bi-daily by nursing staff as part of normal hospital protocol. Demographic and clinical data, including Braden scale scores, BMI, chronic comorbid diagnoses (based on ICD codes), gender, admitting service, and age, were extracted from the electronic medical record. The Braden scale sub-scores most relevant to this study were sensory perception, mobility, and activity, as they directly relate to patient movement and immobility risk. These sub-scores, along with the total Braden score, were included in data analysis. Sub-scores for friction and shear, moisture, and nutrition were excluded, as the sensor system was not designed to assess those domains. Data were cleaned to exclude periods when patients were discharged before sensor removal, identified by prolonged periods of zero variability in sensor angle and pitch measurements at the tail end of recordings. Since normal patient movement resulted in variability in sensor readings, sustained absence of variability was used as a surrogate marker for when the bed was empty following discharge. Permission was obtained to utilize secure hospital wireless networks to support security of patient data.

### Statistical analysis

Patients with <1 hour of sensor recording (*n* = 3) were excluded, resulting in 44 participants in the final analysis. Motion rates were calculated for each patient by dividing total number of movements by time recorded (movements per hour). Patients were categorized as “high movers” or “low movers” based on a threshold of 7.2 movements per hour, the median number of movements per hour for the group, with 22 patients in each group. This threshold was chosen so that each group contained the same number of members. Unpaired t-tests were done in Microsoft Excel comparing average movement rate, age, BMI, and Braden scores between the groups. Next, correlation analyses were performed for the whole sample population that evaluated relationships between movements rate and BMI, and other demographics. A Shapiro-Wilk normality test was performed for each characteristic, which revealed a normal distribution for only age. Movement rate, BMI, number of chronic comorbid diagnoses, and Braden score and sub-scores were not normally distributed. On the basis of non-normally distributed data, Spearman Correlation coefficients (ρ) were calculated in Microsoft Excel to assess the relationship between each characteristic with movement rate. Exact p-values for the Spearman correlation coefficients were calculated in Python (version 13.3.1) using a non-parametric permutation test with 10,000 iterations, in which one variable was randomly permuted and the ρ values were recalculated to generate an empirical null distribution. The two-tailed p-value was defined as the proportion of permuted ρ values as or more extreme than the observed. P-values were calculated as exact due to the non-normality of the data. To estimate 95% confidence intervals, a bootstrapping approach with 1,000 resamples was used, with the 2.5th and 97.5th percentiles of the bootstrap distribution reported as interval bounds. Movement magnitudes were calculated from the vector addition of the x, y, and z components of acceleration from the sensor. Then, we measured the maximum movement magnitude over each 10-minute period. Key statistics, including mean and overall maximum movement magnitude were taken from this data set for each patient to assess how vigorously each patient was moving. Pearson correlation coefficients were calculated in Microsoft Excel between average movement magnitude (the average of each maximal movement intensity for each 10-minute interval), overall maximum movement magnitude, and movements rate. Cluster analysis and heat mapping were performed using Python (version 13.3.1) and associated statistics and visualization libraries, including pandas, sklearn, matplotlib, and seaborn. To identify distinct patient movement patterns, we employed K-Means clustering combined with Principal Component Analysis (PCA) for dimensionality reduction. K-Means is an unsupervised machine learning algorithm that partitions data into non-overlapping groups based on similarity. The optimal number of clusters was determined by calculating silhouette scores across candidate cluster solutions. The number of clusters with the highest silhouette score, 4, was chosen. Given the high-dimensional nature of sensor-derived movement data, PCA was used to reduce the dataset to two principal components, capturing the maximum variance while preserving meaningful structure in the data. The percentage of variance captured by PCA components 1 and 2 was calculated using the PCA model, which measures how much of the total data variation is explained by each component. Motion sensor data, including movement magnitude, movement frequency, angle, and pitch were standardized using z-scores to ensure comparability across features. Cluster analysis was performed on the scaled features to group patients based on movement profiles. Average age, movement rate, BMI, chronic comorbid conditions, Braden scale total score and sub-scores, and admitting team were compared between clusters using unpaired t-tests and Fisher’s Exact Test. For retrospective alert analysis, a Python script (version 3.13.1) was used to identify immobility events, defined as periods of at least 4 continuous hours without detected movement. Additional alerts were generated whenever a patient moved and then experienced another qualifying immobility interval. Sensor data were aggregated into 15-minute time blocks, and a pivot table was created with patient IDs as rows, time blocks as columns, and the average time since the last movement as values. This data was used to generate a heat map visualizing the timing and frequency of simulated immobility alerts across the study population. Comparisons between the alert and non-alert groups and p-values were calculated in Microsoft Excel using unpaired t-tests. All p-values were calculated as two-sided.

## Results

### Sensor validation

**Figure 2.**
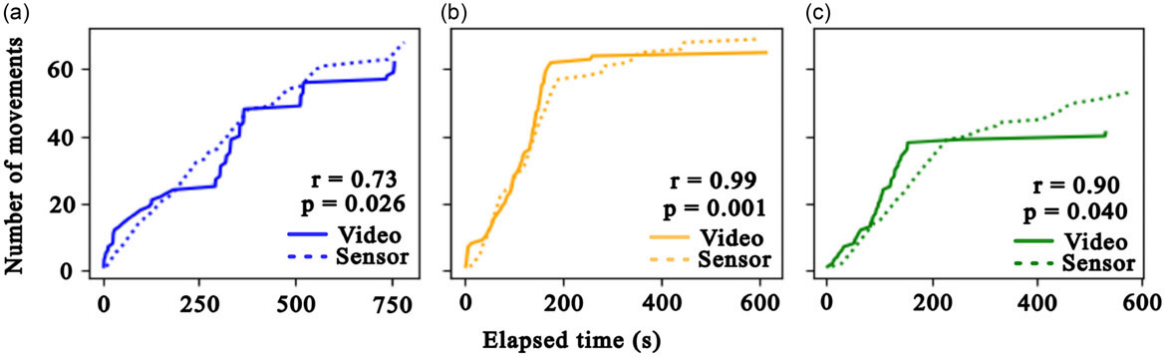
Comparative analysis of motion detection using video recording versus sensor-based measurements in three volunteer subjects. Solid lines represent motion detection based on automated video analysis, while dashed lines represent motion detected by the sensor system. (a–c) motion activity plots for the three individual subjects, showing detected movement over time. The x-axis represents time in seconds, while the y-axis represents number of movements. Pearson correlation coefficients comparing video and sensor measured movements with p-values are shown.

Three volunteers were monitored using video and novel sensor simultaneously during a series of prescribed movements followed by free movement time. There was a strong positive correlation between the movements over time between the videos and sensor (*r* = 0.89, 95% CI [0.51, 0.99]). Figure 2 shows similar patterns in movement and movement rates between the two methods.

### Recording time and movement rates

In the prospective portion of the study, patients were recorded for an average of 22.9 hours with a range of 0.1 hours to 53.4 hours. 44 (94%) patients were recorded for at least 1 hour. These patients had an average movement rate of 9.7 movements per hour with a range of 1.8 to 30.6 movements per hour. Supplementary Figure 1 shows a histogram of recording time and movement rate for the cohort.

### Motion intensity

Motion intensity is a measure of acceleration of the sensor caused by movements. It is a variable of interest in this study, as it helps classify patient movement patterns. It was calculated using the vector sum of the 3 components of acceleration as measured by the sensor, where a_x_, a_y_, and a_z_ represent the components of acceleration in the x, y, and z planes respectively:

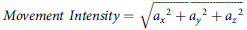




There were significant positive correlations between average movement intensity and movement rate (*r* = 0.51, *p* < 0.001), and total maximum movement intensity and movement rate (*r* = 0.46, *p* = 0.0017).

### Comparison of high movers and low movers

To evaluate whether movement rate correlated with patient characteristics, participants were divided into two equally sized groups based on the median movement rate of 7.2 movements per hour. “High movers” were patients with greater than 7.2 movements per hour (*n* = 22), while “low movers” were patients with movement rates less than 7.2 movements per hour (*n* = 22). Average movement was 15.0 movements per hour for high movers and 4.3 movements per hour for the low movers, which were significantly different. There were no statistically significant differences between the groups for BMI, age, admitting team, comorbid chronic diagnoses, or Braden score and selected sub-scores (Table 2). Supplementary Figure 2 shows (A) a box and whisker plot comparing movement rates between the high and low movement groups, and (B) plots of individual Braden sub-scores (sensory perception, activity, mobility) and total scores, with group means and standard deviations shown.

**Table 2.** High (*n* = 22) vs low (*n* = 22) movement group comparison

Characteristic	Admitting service	High movers	Low movers	*p*
Movement rate (movements/h)		15.05	4.33	<0.001
^a^Sensory perception score		3.86	3.65	0.163
^a^Activity score		2.95	2.52	0.159
^a^Mobility score		3.35	3.05	0.230
Total Braden score		19.69	18.69	0.186
BMI (kg/m^2^)		29.54	27.76	0.295
Age		61.36	65.09	0.515
^b^Number of comorbid chronic diagnoses		8.27	9.59	0.567
Admitting Service	General Medicine	8	12	0.33
	Cardiology	7	7	
	Pulmonology	3	1	
	Surgery	4	1	
	Neurology	0	1	

*Note*: Means shown for continuous variables. Count is presented for categorical variables. *p*-values calculated using Fisher’s Exact Test for categorical variables and unpaired t-tests for continuous variables.

a Sensory perception, activity, and mobility scores are Braden scale sub-scores

b Number of comorbid chronic diagnoses per ICD code.

### Correlation analysis of movement rate and patient-specific factors

Spearman correlation coefficients were calculated for movement rate against several other measures for all patients collectively (Table 3). No significant correlation was found between movement rate and Braden scale scores, BMI, age, or comorbid chronic diagnoses. BMI had the strongest correlation with movement rate (*ρ* = 0.24, *p* = 0.111). Supplementary Figure 3 shows scatterplots for the relationships between movement rate, and BMI, age, number of chronic comorbid conditions, and Braden scores and sub-scores.

**Table 3.** Correlation between movement rate and patient measures

Characteristic	Spearman correlation coefficient	95% CI (lower – upper)	*p*
^a^Sensory perception score	0.21	−0.14 to 0.53	0.171
^a^Activity score	0.12	−0.23 to 0.41	0.442
^a^Mobility score	0.15	−0.16 to 0.46	0.336
Total Braden score	0.16	−0.16 to 0.47	0.301
BMI (kg/m^2^)	0.24	−0.06 to 0.50	0.111
Age	−0.19	−0.19 to 0.11	0.210
^b^Number of comorbid chronic diagnoses	−0.12	−0.12 to 0.17	0.434

*Note*: Correlation coefficients are Spearman correlation coefficients. Correlations are between movement rate (movement per hour) and each measure in the table. CI = confidence intervals.

a Sensory perception, activity, and mobility scores are Braden scale sub-scores.

b Number of comorbid chronic diagnoses per ICD code.

### Cluster analysis

Patients were clustered into groups using the K-Means algorithm, an unsupervised machine learning algorithm that partitions data into non-overlapping groups based on similarity, revealing 4 distinct clusters. The first two principal components explained 37.48% and 22.05% of the total variance, respectively, and were used for visualization in Supplementary Figure 4. Cluster 1 (*n* = 13), cluster 2 (*n* = 28), and cluster 4 (*n* = 2) showed no statistically significant differences in movement rate, BMI, age, or Braden score and selected sub-scores. More patients in cluster 1 were admitted to the Cardiology service compared to cluster 2, 10 vs 2, though this was not a statistically significant difference (*p* = 0.28). The clusters did have a near significant difference in number of chronic comorbid conditions (*p* = 0.052), with an average of 6.1 chronic comorbid diagnoses in cluster 1 and 10.4 chronic comorbid diagnoses in cluster 2. There were no other significant differences between admitting groups for these clusters.

### Alert simulation

**Figure 3.**
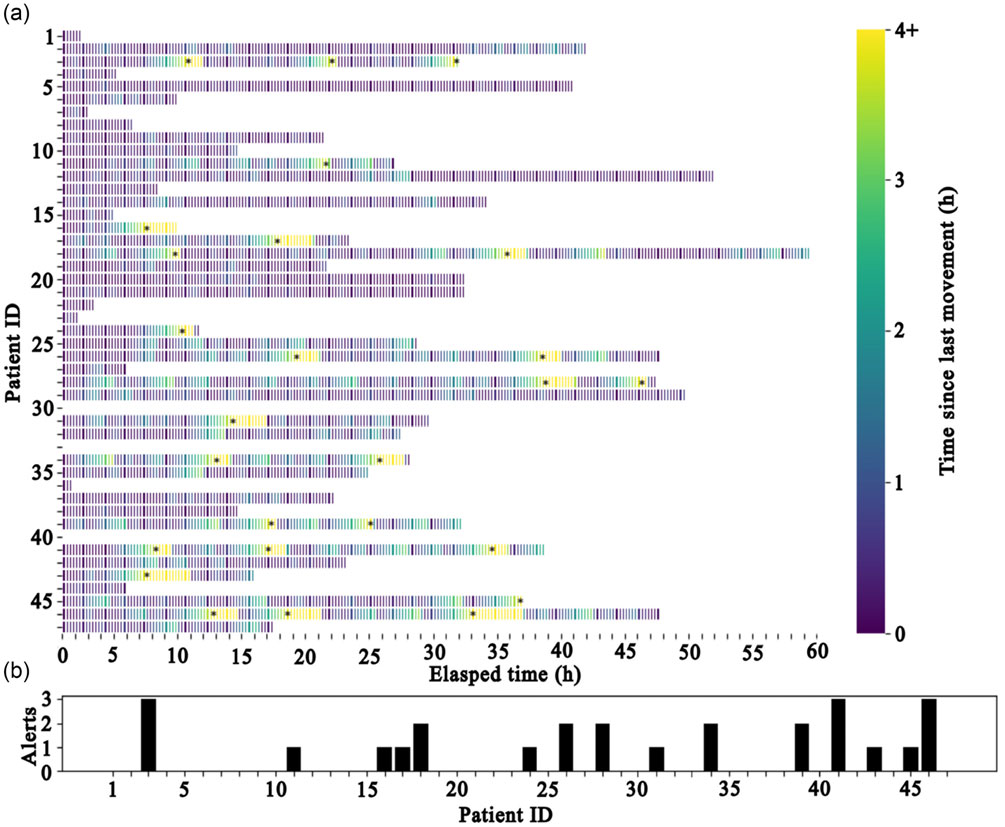
Immobility heat map with simulated alerts. (a) temperature heat map showing time since last movement with elapsed time in 15-minute blocks. Asterisks (*) show where alerts are generated for each patient. (b) number of simulated alerts generated for each patient.

Simulated alerts were generated retrospectively for each patient based on their movement data. An alert was generated if a patient did not move for more than 4 hours. Alerts were generated for 15 of 44 patients (34.1%), predominantly patients with lower movement rates and lower BMIs (Table 4). A heat map was generated to visualize immobility events for each patient. It shows periods of prolonged immobility highlighted in yellow. There were between 1 and 3 alerts generated for these 15 patients, 8 of which had more than 1 alert generated (Figure 3). There was no difference between age, Braden score, or comorbid chronic diagnoses between the patients for which alerts were generated vs those for which alerts were not generated (Table 4). None of the patients in the study developed a pressure injury during their hospitalization.

**Table 4.** Characteristics of patients for which alert was generated (*n* = 15) vs not generated (*n* = 29)

		Alert group	No alert group	
Characteristic	Admitting service	Mean (SD) or count (%)	Mean (SD) or count (%)	*p*
Age		67.9 (24.1)	60.8 (15.4)	0.316
BMI (kg/m^2^)		25.6 (4.1)	30.2 (7.7)	0.012
Movement rate (movements/h)		4.1 (1.8)	12.6 (6.8)	<0.001
^a^Sensory perception score		3.5 (0.5)	3.8 (0.4)	0.065
^a^Activity score		2.6 (1.0)	2.8 (0.7)	0.403
^a^Mobility score		3.0 (0.6)	3.3 (0.6)	0.140
Total Braden score		18.5 (2.5)	19.5 (1.9)	0.165
^b^Number of comorbid chronic diagnoses		10.1 (6.8)	8.3 (6.4)	0.397
Admitting service	General medicine	8 (53.3%)	12 (41.3%)	0.93
	Cardiology	5 (33.3%)	9 (31.0%)	
	Pulmonology	1 (6.7%)	3 (10.3%)	
	Surgery	1 (6.7%)	4 (13.7%)	
	Neurology	0 (0%)	1 (3.4%)	

Note: Means and standard deviations (SD) are shown for continuous variables. Count and percentage relative to total cohort (%) are presented for categorical variables. *p*-values were calculated using unpaired t-tests for continuous variables and Fisher Exact Test for categorical variables. Sensory perception, activity, and mobility scores are Braden scale sub-scores.

a Sensory perception, activity, and mobility scores are Braden scale sub-scores.

b Number of comorbid chronic diagnoses per ICD code.

## Discussion

This study demonstrates the feasibility of implementing a novel bedside motion sensor as a tool for continuous, non-invasive mobility monitoring in hospitalized patients. Despite the small sample size, the findings underscore several critical insights regarding implementation of this technology. Building upon a strong validation phase, where the sensor showed excellent correlation with video-based analysis, the prospective data collection highlights the potential for this technology to supplement traditional nursing assessments and provide real-time mobility data that may enhance pressure injury prevention strategies.

From a translational perspective, this study offers key insights into the operational challenges of deploying a novel medical device in an active clinical setting. While most patients were successfully recorded for extended durations, three were excluded due to early termination of data collection, and an additional five patients had recordings of less than five hours. These failures were caused by sensor disconnection, battery failure, or device removal by staff. An additional unforeseen issue was the hospitals Wireless Intrusion Prevention System, which inhibited the Bluetooth connection between the bed attached sensor and nearby data collection device. These issues emphasized the importance of multidisciplinary education, streamlined workflows, and technical robustness when implementing new technologies in the inpatient environment. These translational challenges mirror those observed in other studies implementing new technology in the hospital setting where device performance, connectivity, and staff training influenced success [23]. Future iterations of this system will prioritize longer battery life, improved wireless data syncing, and clearer staff education to ensure reliability and minimize unintentional disruptions.

Importantly, this study reinforces prior concerns regarding subjectivity and variance between providers of Braden scale assessments [16, 24, 25]. No correlation was found between Braden mobility scores and actual movement rate, and no significant difference in Braden scores was seen between high and low movers. These findings suggest that the Braden scale may not reliably reflect true mobility patterns and that subjective scoring tools may miss at-risk patients due to variability in training, patient presentation, or documentation timing. Integrating sensor-derived data could strengthen risk stratification and decision-making.

One important finding related to sensor performance was the positive correlation between BMI and movement rate, with lower BMI patients exhibiting lower rates of detected movement. One possibility is that patients with lower BMI may have lower baseline mobility due to underlying frailty. This interpretation is supported by prior research from Heyland et al., who found that patients with lower BMI were more likely to experience muscle wasting and functional decline [26]. Alternatively, sensor sensitivity may be impacted by body habitus as larger shifts in mass may result in more detectable mattress deflection, whereas movements in lower-weight individuals may register below the device’s movement threshold. Several studies have demonstrated that BMI can significantly impact sensor performance across a range of technologies, including motion detectors, heart rate monitors, continuous glucose monitors, and pedometers [27–29]. These findings suggest that mobility algorithms may benefit from calibration based on patient BMI to optimize detection accuracy across diverse patient populations. Incorporating such refinements may improve the precision of movement-based alerts and further personalize pressure injury prevention strategies. However, due to the lack of statistical significance of this correlation, these findings should be interpreted with caution and require further investigation in larger, powered cohorts. Motion intensity, calculated from acceleration data, also correlated with overall movement rate and offers a promising additional dimension to future alert systems. High magnitude movements may be more clinically relevant than frequent, small movements, particularly in identifying effective repositioning events. Including both movement frequency and intensity in future alert thresholds may help optimize sensor sensitivity and specificity.

A major translational milestone in this study was the simulation of immobility alerts. These alerts were triggered when patients did not move for more than four hours, consistent with standard hospital repositioning protocols. Sensitivity analysis investigating alternative immobility thresholds was not performed but are planned for future work to better optimize alert performance. Alerts occurred more frequently in patients with lower movement rates and lower BMI. Alerts would have been generated for fourteen patients. This is a high proportion of patients in this study and raises the question of sensor oversensitivity. Excessive or unnecessary alerts could increase the burden on nursing staff, contribute to alert fatigue, and undermine trust in the system, ultimately diminishing its effectiveness. Studies have shown that frequent or non-actionable alerts may reduce caregiver responsiveness [30]. A balance must be achieved between sensitivity and clinical relevance. One contributing factor may be the device’s current inability to detect when a patient is out of bed; patients who were ambulatory for extended periods could have inadvertently triggered alerts despite being at low risk for pressure injury. This limitation is addressed further below. To mitigate the risk of excessive or non-actionable alerts, future iterations of the sensor algorithm may include reduced thresholds for movement detection (e.g. 3 degrees instead of 2 degrees), BMI in the calibration of the algorithm, and bed-exit detection.

Additionally, cluster analysis identified a group of patients with similar movement profiles, predominantly admitted to the cardiology service. This unexpected finding may suggest underlying condition-specific mobility patterns detectable by the sensor. Prior studies have shown reduced activity levels in cardiac patients, which may help explain these results [31]. However, this finding was not statistically significant and should be interpreted with caution. Further, there was no difference in admitting teams between the high and low movement groups. Future research with larger cohorts and diagnostic stratification is warranted to determine whether sensor-derived mobility profiles hold clinical relevance for prognosis, risk assessment, or early detection of clinical deterioration (e.g., decompensation, orthopnea-related changes, or seizure activity). These findings raise the exciting prospect that condition-specific mobility signatures could support real-time monitoring of patient status and recovery trajectories.

While this was a feasibility study and not powered to evaluate clinical outcomes, the high fidelity of movement tracking support further exploration of this system in pressure injury prevention efforts. Importantly, the device is low-cost, requires minimal setup, and does not come into direct contact with the patient. It offers key advantages over existing technologies such as wearable sensors, pressure mats, and under wheel load cells, which may be uncomfortable, damage skin, or require complex installation. Future directions include development of automated alert delivery systems, integration into nursing workflows, and evaluation of clinical impact through randomized trials. These findings contribute to the growing field of sensor-enabled precision care and suggest a pathway toward scalable, real-time mobility tracking tools that align with value-based care and CMS reimbursement models focused on preventable harms like HAPIs. While this study demonstrates the feasibility and potential utility of a low-cost, mattress-attached motion sensor for mobility monitoring, further validation is required before widespread clinical adoption. Larger studies across diverse hospital settings and patient populations are needed to confirm generalizability and performance. Additionally, integration with electronic health records and alignment with nursing workflows will be essential to ensure practical implementation and sustained clinical impact.

## Limitations

This study has several limitations. The validation phase was conducted with only three healthy volunteers, which is a limited number of patients and may not fully represent the complex movement patterns or physical limitations seen in hospitalized patients. While the strong correlation with video analysis is encouraging, further validation in a larger and more clinically diverse population is needed to confirm the device’s performance in real-world hospital settings. In the clinical cohort phase, the sample size was relatively small and limited to a single academic medical center, which may affect the generalizability of the findings. Patient movement was recorded over variable durations, and although most participants were recorded for extended periods, some had limited data due to technical issues such as battery depletion, device dislodgement, or wireless connectivity interference. Additionally, the sensor cannot detect when patients exit the bed. For individuals who spent most of their time out of bed, the movement data may not accurately reflect overall mobility. Future iterations of the device should address this limitation, potentially by incorporating features that recognize bed-exit patterns to pause immobility timers. Movement data were collected continuously, including during sleep, a period of naturally reduced activity. Patients who were predominantly recorded during nighttime hours may have appeared less mobile than they were overall. However, immobility during sleep is recognized as a risk factor for pressure injury development, and regular repositioning during nighttime hours is recommended as a preventative measure [32].

## Supporting information

10.1017/cts.2025.10110.sm001 Smith and Steckler supplementary material Smith and Steckler supplementary material

## Supplementary material

The supplementary material for this article can be found at https://doi.org/10.1017/cts.2025.10110.
