# Supplementary material for: Feasibility and validation of a novel mobility monitoring sensor in hospitalized patients: A prospective cohort study
Source: J Clin Transl Sci. 2025 Jul 24;9(1):e171. doi: 10.1017/cts.2025.10110 (PMC12444695; doi:10.1017/cts.2025.10110)
Supplement: Smith and Steckler supplementary material [file S2059866125101106sup001.docx]

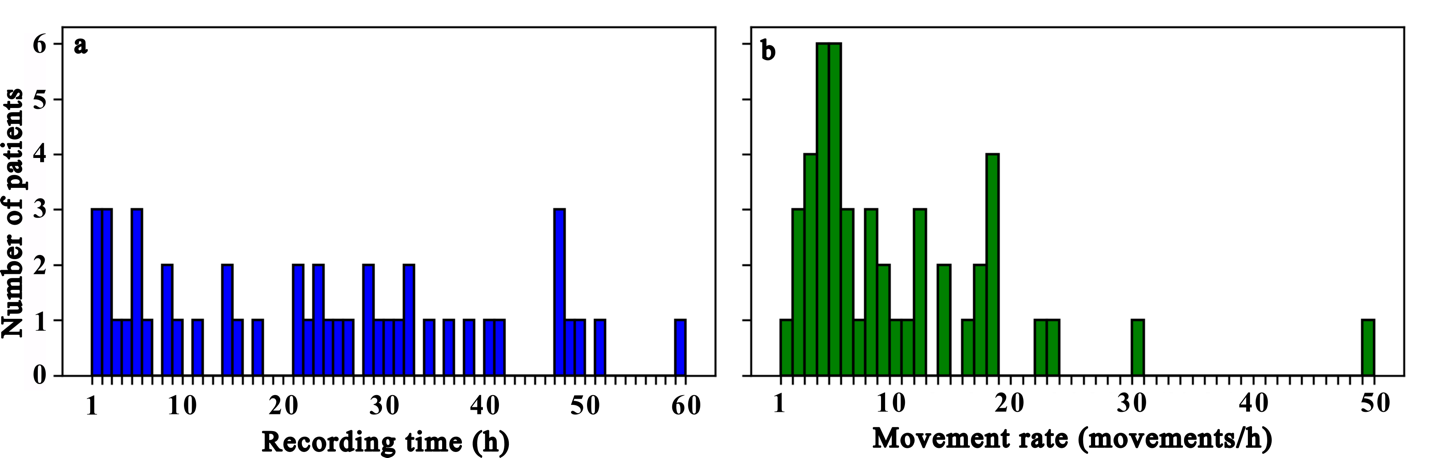


Supplementary figure 1. Recording time and movement rate histograms. (A) Histogram of recording time in hours for all patients (n = 47). Patients were recorded for varying durations ranging from 0.1 to 53.4 hours, with 44 patients recorded for > 1 hour. (B) Histogram of movement rate in movements per hour for all patients (n =47), movement rates ranged from 1.8 to 50.4 movements per hour, with most patients falling between 2 and 20 movements per hour.


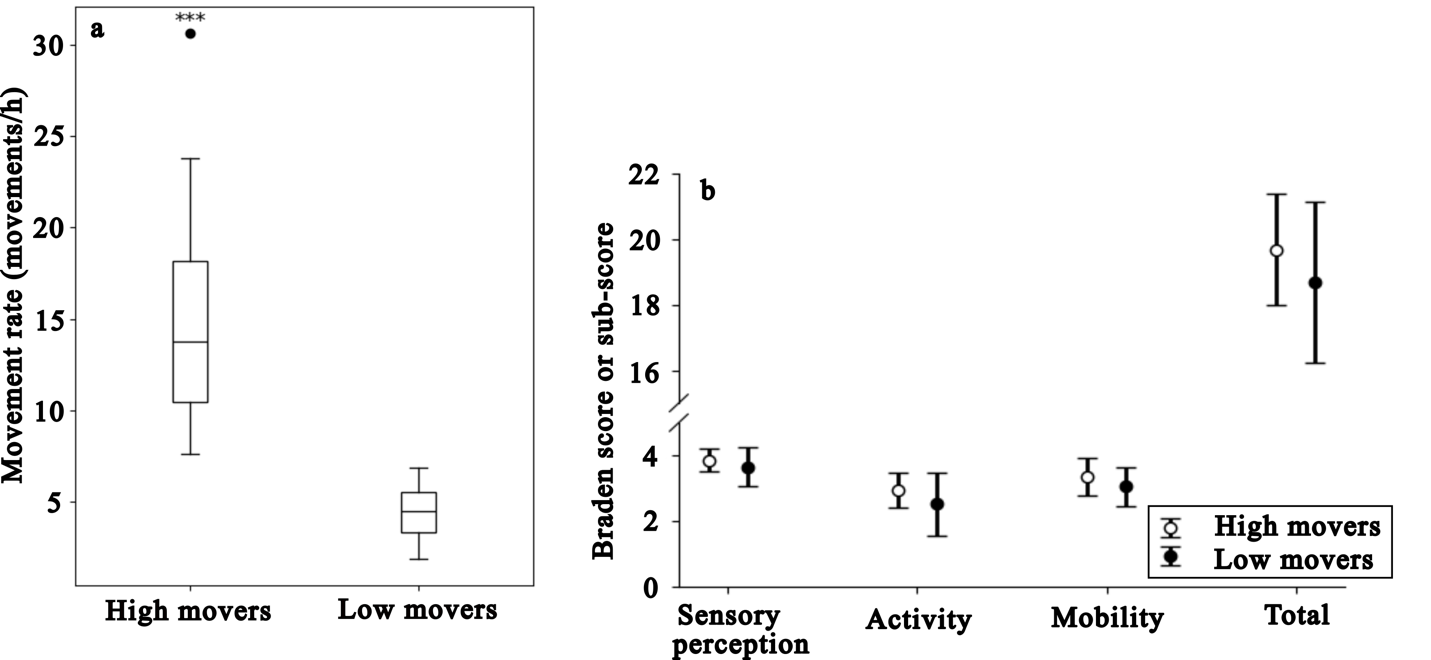


Supplementary figure 2. High and low movement group comparisons. (A) Box and whisker plot comparing movement rate between the high (n = 22) and low (n = 22) movement groups. (B) Average Braden scores compared between the high and low movement groups. Sensory perception, activity, and mobility are Braden scale sub-scores. “Total” is total Braden scale score. Data are means +/- standard deviation. *** p<0.001.


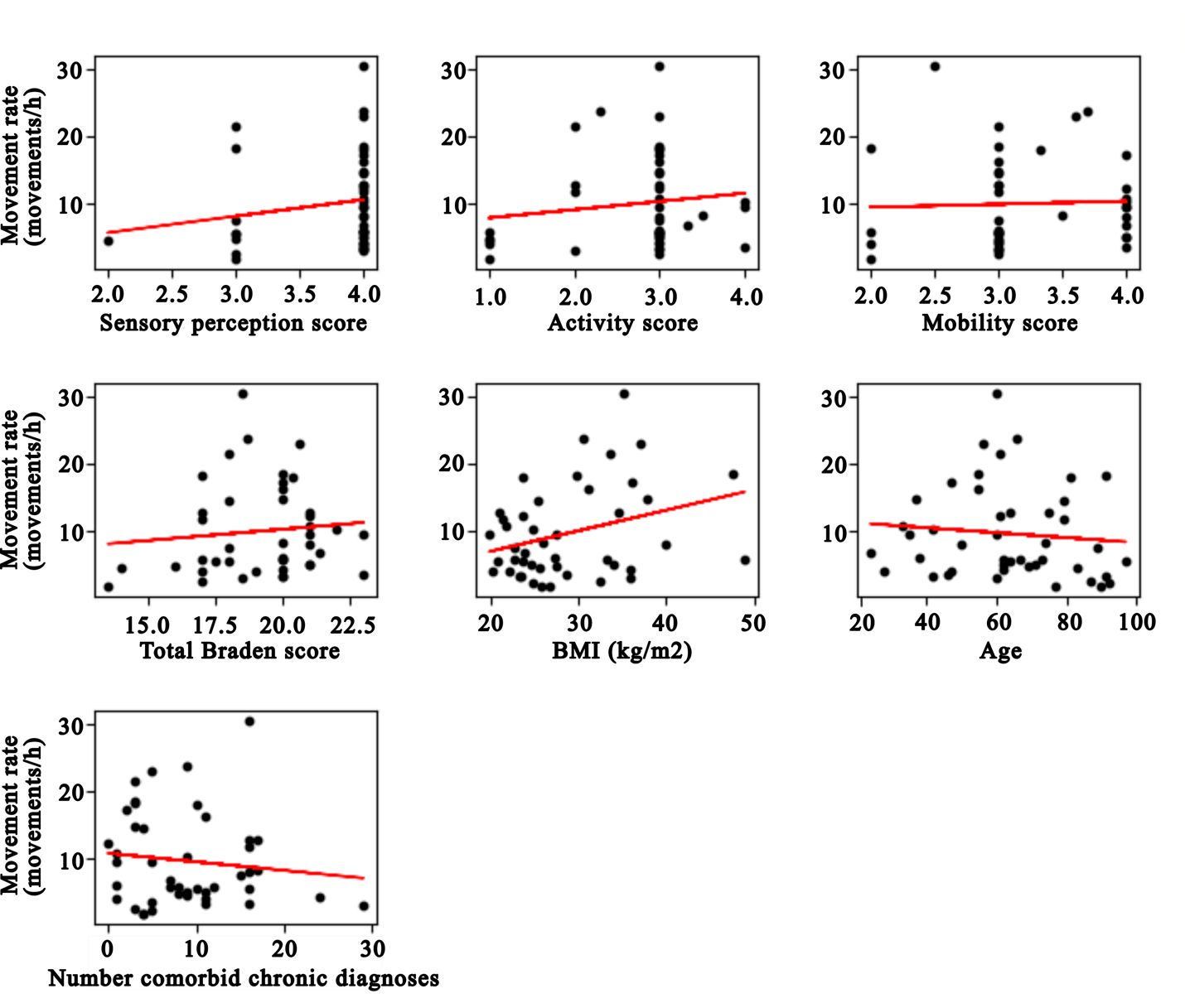


Supplementary figure 3. Scatter plots showing the relationship between movement rate (movements per hour) and individual patient characteristics, including BMI, Braden total score, and Braden sub-scores (sensory perception, activity, and mobility). Each point represents one participant (n = 44). Axes are scaled individually for each plot to optimize visual clarity.


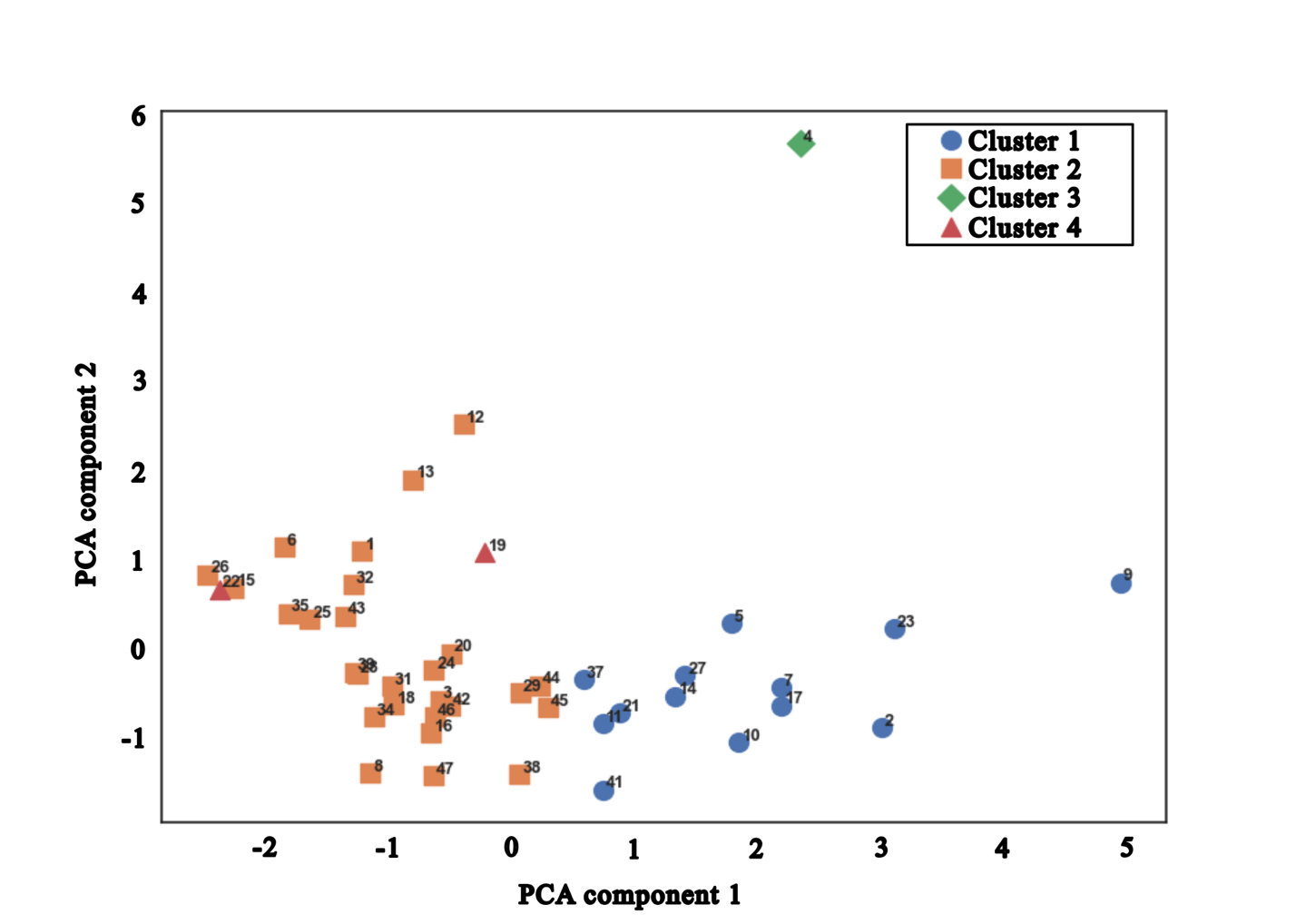


Supplementary figure 4. K-means clustering (PCA plotted). Clusters (n = 4) represented by different colored data points. Each data point is labeled with the corresponding patient ID.
